# Taking U out, with two nucleases?

**DOI:** 10.1186/1471-2105-7-305

**Published:** 2006-06-16

**Authors:** I Saira Mian, Elizabeth A Worthey, Reza Salavati

**Affiliations:** 1 Life Sciences Division, Lawrence Berkeley National Laboratory, Berkeley, California 94720-8265, USA; 2 Seattle Biomedical Research Institute, Seattle, Washington, 98109, USA; 3 McGill University, Institute of Parasitology, Ste.-Anne-De-Bellevue, Quebec, H9X 3V9, Canada

## Abstract

**Background:**

REX1 and REX2 are protein components of the RNA editing complex (the editosome) and function as exouridylylases. The exact roles of REX1 and REX2 in the editosome are unclear and the consequences of the presence of two related proteins are not fully understood. Here, a variety of computational studies were performed to enhance understanding of the structure and function of REX proteins in *Trypanosoma *and *Leishmania *species.

**Results:**

Sequence analysis and homology modeling of the Endonuclease/Exonuclease/Phosphatase (EEP) domain at the C-terminus of REX1 and REX2 highlights a common active site shared by all EEP domains. Phylogenetic analysis indicates that REX proteins contain a distinct subfamily of EEP domains. Inspection of three-dimensional models of the EEP domain in *Trypanosoma brucei *REX1 and REX2, and *Leishmania major *REX1 suggests variations of previously characterized key residues likely to be important in catalysis and determining substrate specificity.

**Conclusion:**

We have identified features of the REX EEP domain that distinguish it from other family members and hence subfamily specific determinants of catalysis and substrate binding. The results provide specific guidance for experimental investigations about the role(s) of REX proteins in RNA editing.

## Background

Most mitochondrial mRNAs in trypanosomatid parasites such as *Trypanosoma*, and *Leishmania species *undergo RNA editing [1-3]. This post-transcriptional process produces mature and functional mRNAs through a series of coordinated steps catalysed by a multi-protein complex that inserts and deletes uridylates (Us) specified by guide RNAs (gRNAs). One hypothesis posits a structural and functional subdivision of the editosome into insertion and deletion subcomplexes [4-8]. Editosome proteins with endonuclease (REN1, REN2) [9,10], terminal uridylyl transferase (TUTase; RET1, RET2) [6,11,12], 3' exouridylylase (exoUase; REX1, REX2[5,13], Ernst *et al*., unpublished), ligase (REL1, REL2) [5,8,14,15], and helicase (REH1) [16] activities have been identified and functionally characterized. Sets of proteins related by sequence similarity exhibit both unique and common functions. For instance, REN1 is an endoribonuclease that is specific for RNA editing deletion sites whereas REN2 is specific for RNA editing insertion sites. RET1 is implicated in the addition of the non-encoded 3'-oligo U tails to gRNAs but RET2 adds Us to pre-edited mRNAs. REL1 may be involved in U-deletion editing and REL2 in U-insertion editing. Six additional editosome proteins, KREPA1-A6, have varying degrees of sequence relatedness with each protein containing a C-terminal motif associated with an oligonucleotide-binding (OB) fold [5,17-20]. Recent results point to both REX1 and REX2 as candidates for the RNA editing exoUase responsible for deletion of the 3' overhanging U residues from the mRNA 5' cleavage fragment. A U-specific exonuclease, REX1, has been partially purified from *L. tarentolae *[13]. The reconstitution of precleaved U-deletion *in vitro *with recombinant *L. tarentolae *REX1 and REL1 proteins and the *in vivo *RNAi down-regulation of REX1 expression in *T. brucei *suggest that REX1 is the exoUase. However, the closely related REX2 protein (28% overall identity and 46% similarity in *T. brucei*) may be the putative exoUase since tagged *T. brucei *REL1 sub-complex consisting of REX2, REL1 and KREPA2 catalyze accurate U removal and ligation (i.e. pre-cleaved deletion editing) [5]. Thus, the exact roles of REX1 and REX2 in the editosome complex are unclear and the consequences of the presence of two related proteins are not fully understood.

Comparative sequence analysis indicates that both REX1 and REX2 contain a putative C-terminal Endonuclease/Exonuclease/Phosphatase (EEP) domain as well as a region exhibiting subtle, but significant similarity to a known 5'->3' exonuclease domain (*L. major *REX2 lacks an EEP domain because of a truncation at the C-terminus) [17]. Whether REX1 and REX2 have 5'->3' exonuclease activity in the editing complex is unknown.

In this study, we extend our previous analysis of the REX1 and REX2 EEP domains [17]. We use sequence analysis, homology modeling and phylogenetic analysis to enhance understanding of the structure and functions of REX proteins, as well as the relationships amongst EEP family members. Our results suggest that while these enzymes have diverged at the sequence level, the EEP domains share a common catalytic site. Our three-dimensional modeling studies suggest that the REX EEP domains fold in much the same way as other EEP domains whose structures have been determined by X-ray crystallography. We identify features of the REX EEP domain that distinguish it from other family members and hence subfamily specific determinants of catalysis and substrate binding.

## Results and discussion

### Trypanosomal REX proteins

**Figure 1:**
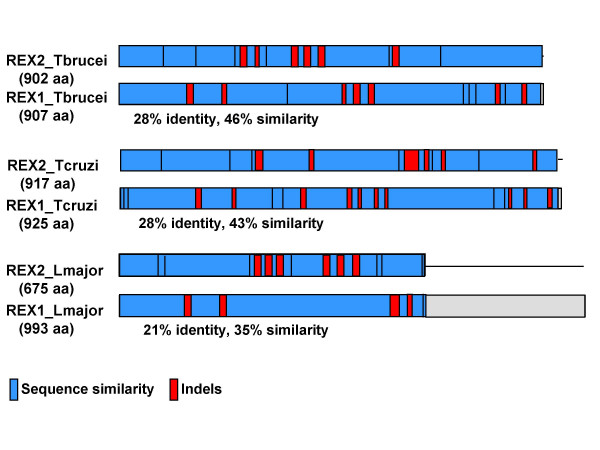
Schematic diagram of the REX1 and REX2 editosome proteins from *Trypanosoma brucei *(REX1_Tbrucei, REX2_Tbrucei), *T. cruzi *(REX1_Tcruzi, REX2_Tcruzi), and *Leishmania major *(REX1_Lmajor, REX2_Lmajor). Each protein is represented as a horizontal bar and the number of amino acids (aa) is given. The percent identity and similarity for each pair of putative paralogs is indicated. The regions of similarity are in blue whereas segments that are added or deleted in one paralog (Indels) are in red. In *L. major*, the C-terminal portion of REX1 which contains an EEP domain (gray) has no counterpart in REX2 because of a truncation.

The REX1 and REX2 proteins from three trypanosomatids show considerable sequence similarity suggesting they are encoded by paralogous genes (Fig. 1). Since the genes are present on non syntenic chromosomal regions in *L. major *and *T. brucei *(chromosome assignments not having been made for the *T. cruzi *genes) it is likely that the ancestral genes diverged prior to the fission/fusion events which resulted in the modern day trypanosomatid genomes.

### Sequence and phylogenetic analysis of EEP domains

**Figure 2:**
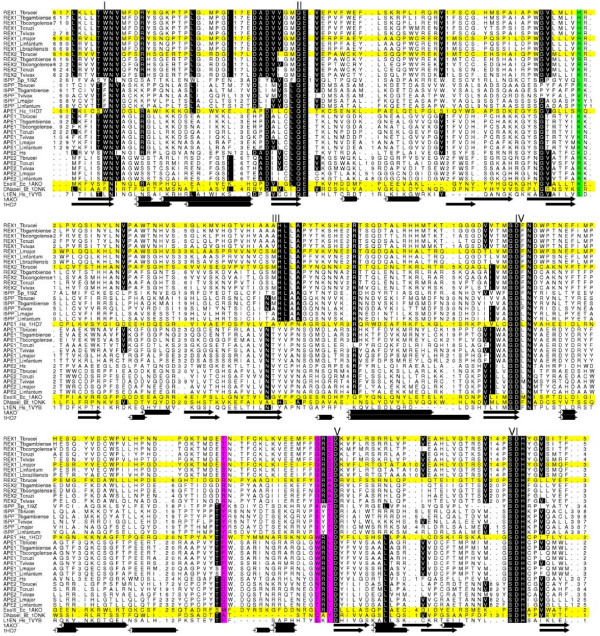
HMM-generated multiple sequence alignment of the EEP domain from a variety of Bacteria and Eucarya. The EEP domain at the C-terminus of REX2 and REX1 is the putative exonuclease component of the editosome. Residues that are conserved in >50% of the sequences are in black and numbers indicate the number of amino acids not shown explicitly. Six EEP domains of particular interest are in yellow. The Roman numerals above the alignment mark the six conserved sequence motifs that have been used to characterize EEP domain (see also Table 1). Columns in pink indicate the putative substrate specificity active-site hydrophobic pocket. The column in green marks the location of the essential Glu in DNaseI (DNaseI_Bt_1DNK, the last sequence highlighted in yellow). The proteins shown are REX1 (RNA editing exonuclease 1), REX2 (RNA editing exonuclease 2), I5PP (inositol polyphosphate 5'-phosphatase), APE1 (apurinic/apyrimidinic endonuclease 1), and APE2 (apurinic/apyrimidinic endonuclease 2); these sequences are from *T. brucei *(Tbrucei), *T. gambiense *(Tbgambiense), *T. congolense *(Tbcongolense), *T. cruzi *(Tcruzi), *T. vivax *(Tvivax), *L. major *(Lmajor), *L. infantum *(Linfantum), *L. braziliensis *(Lbraziliensis), *Schizosaccharomyces pombe *(Sp), *Homo sapiens *(Hs), *Escherichia coli *(Ec), and *Bos taurus *(Bt). EEP domains whose structures have been determined experimentally are I5PP_Sp_1I9Z (*S. pombe *phosphatidylinositol phosphate phosphatase, RCSB code1I9Z), APE1_Hs_1HD7 (*H. sapiens *AP endonuclease 1, 1HD7), ExoIII_Ec_1AKO, (*E. coli *exonuclease III, 1AKO); DNaseI_Bt_1DNK (*B. taurus *deoxyribonuclease I, 1DNK) and L1EN_Hs_1VYB: (*H. sapiens *L1 endonuclease, 1VYB). Cylinders and arrows denote the α-helices and β-strands given in the RCSB entries 1AKO and 1HD7. The protein sequences, EEP domain HMM and alignment are available as Supplementary material.

**Figure 3:**
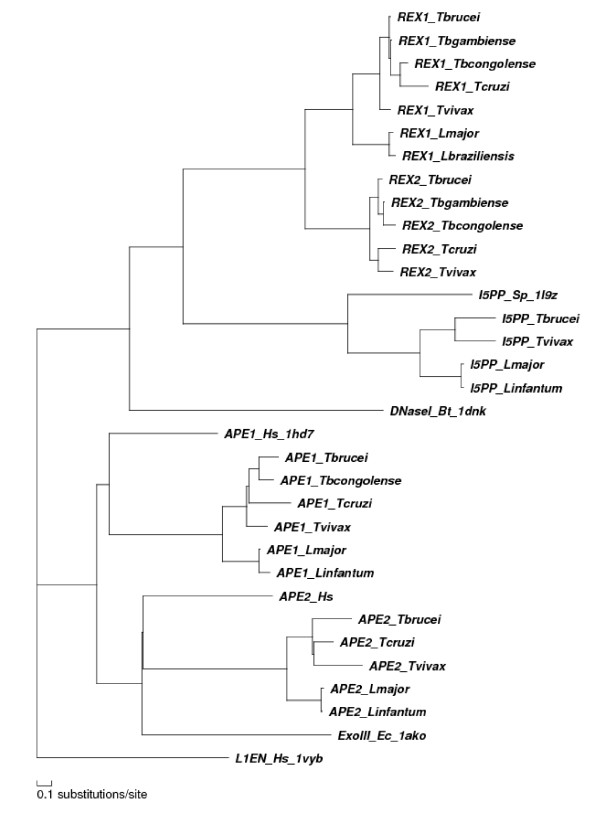
A maximum likelihood tree estimated from an HMM-generated multiple sequence alignment of EEP protein domains (Fig. 2 provides information on the proteins shown).

The EEP domains in proteins from a variety of Eucarya and Bacteria were modelled and analyzed using an HMM-based approach. Members of the EEP domain family include magnesium dependent endonucleases (L1-EN, DNaseI, APE1, APE2) [21-26], exonucleases (ExoIII, REX1, REX2) [5,13,17,27], and phosphatases of lipid second messengers (I5PP) [28] (Fig. 2). Although these proteins have diverse substrate specificities, REX EEP domains possess the conserved sequence motifs that have been used to characterize other EEP domains (I to VI, Fig. 2). A phylogenetic tree of EEP domains indicates that REX and APE proteins form distinct subfamilies (Fig. 3). EEP domains in I5PP proteins are more REX- than APE-like.

### Homology modelling of T. brucei REX1 and REX2 and L. major REX1

The X-ray crystal structures of the EEP domains in two DNA repair enzymes (Fig. 2, APE1_Hs_1HD7, ExoIII_Ec_1AKO) [25,27] were used as the templates to build homology models of the EEP domains in three REX proteins (REX1_Tbrucei, REX2_Tbrucei, REX1_Lmajor). *H. sapiens *APE1 and *E. coli *ExoIII are functional homologs that possess apurinic/apyrimidinic (AP) endonuclease activity and which hydrolyze the phosphodiester bond of DNA at the AP sites by cleaving the DNA in intact strands [29]. Although ExoIII also has 3'->5' exonuclease activity, its biological role remains unclear. Following cleavage by ExoIII or APE1 the bacterium *E. coli *uses DNA polymerase I (pol I) to fill in the single-nucleotide gap whereas the eucaryote *H. sapiens *uses DNA polymerase β (pol β). Pol I has also a 3'->5' proof-reading activity which allows the removal of misincorporated nucleotides [30]. Although pol β is prone to high error (one mistake per 4000 bases inserted) [31], it lacks the proof reading mechanism found in pol I [32]. Instead, APE1 acts as an exonuclease that trims off nucleotides from DNA ends that do not terminate in correct basepairs [33].

**Figure 4:**
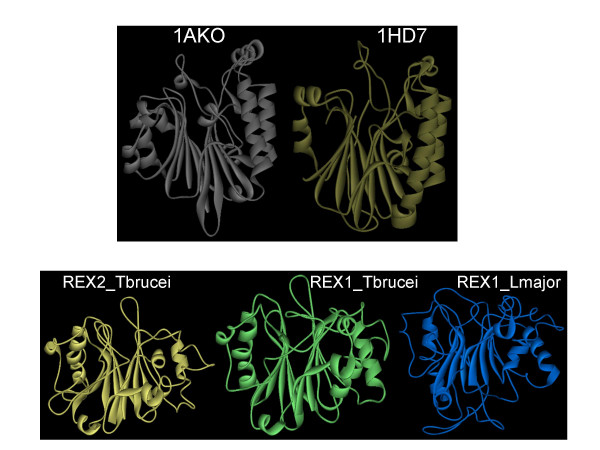
Three-dimensional models of trypanosomal REX EEP domains built using EEP domains whose structures have been determined by X-ray crystallography. The upper panel shows ribbon diagrams for two experimentally determined structures: 1AKO is *E. coli *exonuclease III (Fig. 2, ExoIII_Ec_1AKO) and 1HD7 is *H. sapiens *APE1 (APE1_Hs_1HD7). The lower panel shows ribbon diagrams of the homology-built structures: *T. brucei *REX2 (REX2_Tbrucei), *T. brucei *REX1 (REX1_Tbrucei) and *L. major *REX1 (REX1_Lmajor). The four-layered α, β sandwich fold in each EEP domain is shown in the same orientation with the substrate binding surface at the top.

**Figure 5:**
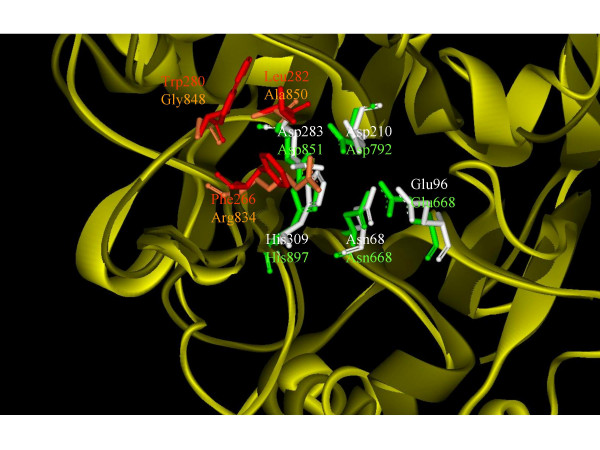
The active sites of APE1_Hs_1HD7 (green) superposed onto REX2_Tbrucei (yellow) with the side chains of critical amino acids shown explicitly. The APE1 (REX2) catalytic residues are in white (green) and the hydrophobic residues are in red (orange).

**Figure 6:**
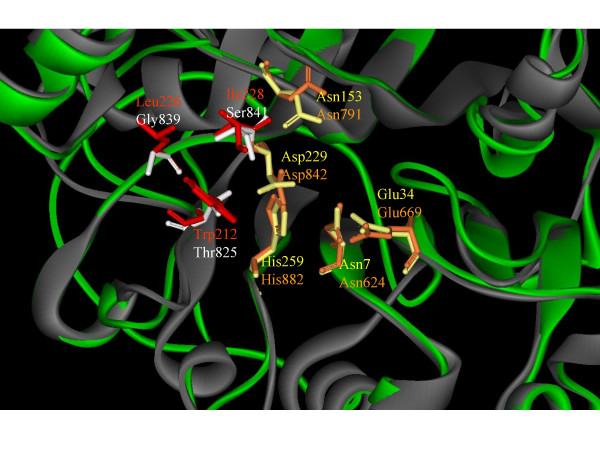
The active sites of ExoIII_Ec_1AKO (grey) superposed on REX1_Tbrucei (green) with the side chains of critical amino acids shown explicitly. The ExoIII (REX1) catalytic residues are in yellow (orange) and the hydrophobic residues are in red (white).

**Figure 7:**
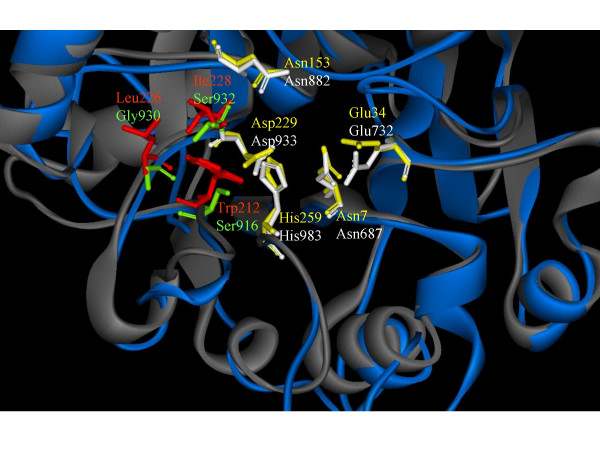
The active sites of ExoIII_Ec_1AKO (grey) superposed on REX1_Lmajor (blue) with the side chains of critical amino acids shown explicitly. The ExoIII (REX1) catalytic residues are in yellow (white) and the hydrophobic residues are in red (green).

As would be expected, the four-layer α/β fold observed in the crystal structures of the templates (APE1/1HD7; ExoIII/1AKO) is reflected in the three-dimensional models of the target proteins (Fig. 4). The roles of amino acids in the conserved sequence motifs found in EEP domains (I to VI, Fig. 2) were examined in APE1/1HD7 and ExoIII/1AKO (Table 1). Based on high resolution X-ray crystal structures, the similar overall catalytic mechanism of APE1 and ExoIII involves the abstraction of a proton from a water molecule by a residue acting as a general base [23,27]. The resultant nucleophilic hydroxide ion attacks a scissile phosphate. The major difference between APE1 and ExoIII is that the catalytic residue which deprotonates the water molecule in APE1 is Asp in motif IV whereas in ExoIII it is the His in motif VI (Table 1 and Fig. 5, 6, 7). In addition, ExoIII appears to be a relatively more powerful 3'-exonuclease than APE1 [34,35]. This enhanced activity has been attributed to the fewer hydrophobic residues in the active site of ExoIII [23,24] (and see below).

### Putative activity and mechanisms of action of REX proteins

To gain insights into the enzymatic activity of the REX proteins, we measured the rmsd values of the superposed models of each of REX EEP domain model to APE1/1HD7 and ExoIII/1AKO. The REX2_Tbrucei model is closer to APE1/1HD7 than to ExoIII/1AKO (2.54 Å versus 4.19 Å). In contrast, both the REX1_Lmajor and REX1_Tbrucei models are closer to ExoIII (2.17 Å, 1.97 Å, respectively) than to APE1/1HD7 (3.41 Å, 3.91 Å, respectively). These results suggest that REX2_Tbrucei may have more in common with APE1/1HD7 whereas REX1_Lmajor and REX1_Tbrucei may be more related to ExoIII/1AKO. The latter data suggest that REX1 exoribonuclease activity in both *Leishmania *and *Trypanosoma *species may share a similar catalytic mechanism with ExoIII [13]. These results also raise the possibility that if REX2_Tbrucei is similar to APE1, the potential proof-reading function of *Trypanosoma *REX2 may remove (i) the extraneous U residues added during TUTase activity [36-38] and (ii) the Us that result from TUTase function within a U-deletion site [39]. Current data indicate that *Leishmania *REX2 lack a C-terminal EEP domain (Fig. 1, Fig. 2) and hence potential proof-reading activity. Although this function could be compensated for by the related REX1 protein (see below), the absence of an EEP domain could be explained by less extensive editing in *Leishmania *species compared to *Trypanosoma *family members. While both APE1 and ExoIII are known to act as endonucleases, such activity has not yet been demonstrated for the REX family of proteins.

In addition to the primary catalytic residues, the active site of APE1/1HD7 and ExoIII/1AKO contains a bulky hydrophobic pocket that has been proposed to act as a sequence specific "gate-keeper" able to accommodate only abasic sites [23,24]. In APE1/1HD7, the hydrophobic pocket is composed of Phe266, Trp280, and Leu282 (Fig. 2, Fig. 5). The equivalent pocket in ExoIII/1AKO is larger and consists of Trp212, Leu226, and Ile228 (Fig. 2, Fig. 6, 7). Mutations of the APE1 hydrophobic pocket that results in smaller residues (e.g. Phe266 to Ala/Cys, Trp280 to Ile/Leu/Ser), can enhance its 3'-exonuclease activity [24]. ExoIII possesses a hydrophobic pocket containing only one aromatic residue and the enzyme is a better 3' exonuclease than APE1. These two findings support the idea that the hydrophobic pocket in EEP domains plays a significant role in nucleotide binding and specificity. Our sequence and structural analysis suggests that REX1 and REX2 do not have a bulky hydrophobic pocket but instead share a pocket composed of smaller residues. The pocket is formed by Arg834, Gly848, and Ala850 in REX2_T.brucei, Thr825, Gly839, and Ser841 in REX1_Tbrucei, and by Ser916, Gly930, and Ser932 in REX1_Lmajor (Fig. 2, Fig. 5, 6, 7). The equivalent of Trp280 in APE1/1HD7 or Leu226 in ExoIII/1AKO is Gly839 in REX1_Tbrucei, Gly848 in REX2_Tbrucei, and Gly930 in REX1_Lmajor. Thus, we predict that REX1 and REX2 have the potential to accommodate an extrahelical residue (i.e. uridine) downstream (3') of the scissile bond. The conserved polar Ser/Thr in REX1, and positively charged Arg in REX2 may form hydrogen bonds with the extrahelical base (column in pink between conserved motifs IV and V in Fig. 2). In REX1, a Ser in *Leishmania *is a Thr in *Trypanosoma *(Fig. 6, 7). This suggests altered substrate specificity for *Leishmania *species, which may partially compensate for the absence of an EEP domain in REX2.

Comparison of the REX subfamily of EEP domains with that of the *B. taurus *DNase I (DnaseI_Bt_1DNK, Fig. 2) and inositol polyphosphate 5'-phosphatase (I5PP, Fig. 2) family members reveals a conserved His in motif III (Fig. 2 and Table 1) [22,28,40]. In DNase I, this residue is part of the essential His-Glu catalytic pair located within the active site and is proposed to act as a general acid acting to stabilize the leaving group [22]. Mutagenesis of Glu in DNase I has also shown the importance of this residue for catalytic activity. However, the sequence analysis of the REX and other family members of EEP domains does not reveal a conserved Glu or other negatively charged residue that could pair with the His residue (Fig. 2) [41]. Therefore, we predict that in the REX EEP domains, the His residue forms a hydrogen bond to and further polarizes the scissile phosphate, as previously proposed for the I5PP family of proteins [41].

## Conclusion

Using a variety of computational approaches, we have identified conserved motifs and a critical substrate binding pocket in the REX subfamily of EEP domains. Our results suggest experiments that could be performed to examine the distinct catalytic roles of REX proteins in the editosome.

## Methods

### Trypanosomal proteins

The genomic locations of the trypanosomal proteins discussed in this work were determined using the GeneDB Artemis interface to data from the TriTryp genomes sequencing consortium [42]. Putative orthologs were initially identified using BLAST searches of the unfinished genome sequences. These findings were later confirmed through mutual best BlastP analysis amongst the unique portions of the three essentially complete trypanosomatid genomes. The sequences of these homologous genes were also confirmed against the high coverage sequences available from the genome projects. The available, unfinished genome sequences for the remaining trypanosomatids discussed in this manuscript have been made available through GeneDB, and were searched using the blast algorithm to identify putative orthologues in these species. In a number of instances, the matches were not full length, and the gene was present in more than a single contig, thereby requiring assembly of the sequence to obtain full-length genes.

### Sequence and phylogenetic analysis of EEP domains

The sequence and phylogenetic analysis of EEP domains was performed using a hidden Markov model (HMM)-based approach that has been employed successfully elsewhere (see, for example, the following refs [43-47]).

Previously, we estimated an HMM of the EEP domain using the SAM software suite version 3.3.1 [48] and a limited number of protein sequences [17]. For this work, the parameters of this initial HMM were updated using an expanded training set that included additional eukaryotic (including trypanosomatid) and bacterial sequences. The ensuing EEP domain HMM was used to generate a multiple sequence alignment of all the EEP domains in the training set and the alignment was annotated with known structural information for some members of the EEP domain family (Fig. 2).

A phylogenetic tree for EEP domains was estimated using an HMM-generated multiple sequence alignment of the training set and ProtML in the MOLPHY software suite version 2.3b3s. Since insert states in the HMM are uninformative, the alignment consisted only of residues aligned to match states of the EEP HMM. ProtML infers an evolutionary tree from amino acid sequences using the Maximum Likelihood (ML) method. The tree with the maximum likelihood was used to understand the relationships between EEP domains.

### Homology modeling

Three-dimensional models of selected REX EEP domains were built as described previously [49] using the MODELLER program [50] using software programs from Accelrys Inc., DS Modeling 1.1 and an alignment of a domain of unknown structure against a domain of known structure (Fig. 2). The sequences/structures of APE1_Hs_1HD7 and ExoIII_Ec_1AKO were used as the templates for constructing models of three targets, REX1_Tbrucei, REX2_Tbrucei and REX1_Lmajor. This particular choice was based on (i) the functional homology and multiple sequence alignment (Fig. 2), (ii) a statistically significant PSI-BLAST score between the target and an EEP family protein (E-value = 5e-12 REX1_Tbrucei, 6e-08 REX1_Lmajor, and 2e-08 REX2_Tbrucei), and (iii) a statistical significant score produced by 3D-Jury (120–156, well above the cutoff value of 50). The 3D-Jury metaserver [51] selects the most abundant models from the set of 3D models generated by various independent prediction providers. To measure the r.m.s. deviation of the superposed template and the target, the complete sequences of the predicted EEP domains aligned in Figure 2 were used to measure the  r.m.s. deviation values. The quality of predicted modeled structures were checked with the Profiles_3D program [52] in DS Modeling 1.1.

## Abbreviations

REX, RNA editing exouridylylase; EEP, Endonuclease/Exonuclease/Phosphatase; REN, RNA editing endonuclease; RET, RNA editing terminal uridylyl transferase; TUTase, terminal uridylyl transferase; exoUase, exouridylylase; REL, RNA editing ligase; REH, RNA editing helicase, APE, apurinic/apyrimidinic endonuclease; OB, oligonucleotide binding; L1-EN, L1 endonuclease; ExoIII, exonuclease III; I5PP, inositol polyphosphate 5'-phosphatase; pol I, DNA polymerase I;pol β, DNA polymerase β.

## Authors' contributions

ISM carried out the HMM analysis and phylogenetic inference of the EEP domains and contributed to writing the manuscript. EAW identified the EEP domain homologs and contributed to the phylogenetic inference analyses. RS carried out the homology modeling analysis, drafted the manuscript, conceived and coordinated the study. All authors read and approved the final manuscript.

## Figures and Tables

**Table 1:** Conserved sequence motifs found in EEP domains (labeled I through VI in Fig. 2) and the roles of specific amino acids in two EEP domains of known three-dimensional structure (APE1_Hs_1HD7, ExoIII_Ec_1AKO in Fig. 5–7).

REX1/REX2	APE1	EXO III
I. TWN	Asn forms a hydrogen bond to catalytic Asp in motif IV	Asn interacts with 5' phosphate group
II. QE	Glu coordinates Mg^2+ ^ion	Glu coordinates Mg^2 ^ion
III. HL	His is substituted by Tyr in APE1 but is the catalytic His in DNase I family	His is substituted by Tyr
IV. GDFN	Catalytic Asp deprotonates the water molecule; Asn forms a hydrogen bond to scissile phosphate	Asn forms a hydrogen bond to scissile phosphate
V. GRXD (X is S in REX1 and A in REX2)	Asp forms a hydrogen bond to His in motif VI	Asp forms a hydrogen bond to His in motif VI
VI. SDH	His forms a hydrogen bond to Asp in motif V and to scissile phosphate	Catalytic His deprotonates the water molecule and forms a hydrogen bond to Asp in motif V
